# Climate change differentially alters distribution of two marten species in a hybrid zone

**DOI:** 10.1002/ece3.70181

**Published:** 2024-08-19

**Authors:** Helen E. Chmura, Lucretia E. Olson, Remi Murdoch, Alexandra K. Fraik, Scott Jackson, Kevin S. McKelvey, Rex Koenig, Kristine L. Pilgrim, Nicholas DeCesare, Michael K. Schwartz

**Affiliations:** ^1^ Wildlife Ecology Program, Rocky Mountain Research Station U.S. Forest Service Missoula Montana USA; ^2^ U.S. Forest Service Northern Regional Office Missoula Montana USA; ^3^ Hellgate High School Missoula Montana USA; ^4^ Montana Fish, Wildlife and Parks Missoula Montana USA

**Keywords:** American marten, climate change, distribution, hybridization, maximum entropy, Pacific marten

## Abstract

Species' ranges are shifting rapidly with climate change, altering the composition of biological communities and interactions within and among species. Hybridization is among the species interactions that may change markedly with climate change, yet it is understudied relative to others. We used non‐invasive genetic detections to build a maximum entropy species distribution model and investigate the factors that delimit the present and future ranges of American marten (*Martes americana*) and Pacific marten (*Martes caurina*) in a contact zone in the Northern Rockies. We found that climate change will decrease the suitable habitat predicted for both species, as well as the amount of overlap in predicted suitable habitat between the species. Interestingly, predicted suitable habitat for Pacific marten extended further north in the study region than our genetic detections for the species, suggesting that biotic factors, such as interactions with American marten, may affect the realized range of this species. Our results suggest that future work investigating the interactions among biotic and abiotic factors that influence hybrid zone dynamics is important for predicting the futures of these two species in this area under climate change.

## Abstract 2

Climate change is altering the distributions of species worldwide, including those of hybridizing species. We use species distribution models, built from non‐invasive genetic detections of marten species, to show that the distributions of American marten and Pacific marten in the Northern Rockies of the United States will be differentially affected by climate change.

## Graphical abstract

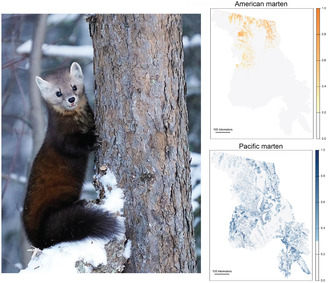

## 1 INTRODUCTION

Numerous studies document species' range shifts with global climate change (e.g., Chen et al., 2011; Lenoir & Svenning, 2015; Parmesan & Yohe, 2003). Climate change can influence species ranges' directly, by altering local climatic suitability, as well as indirectly, by altering the biotic factors that influence species' ranges (Thomas, 2010). When range shifts are asymmetric across species, they change the composition of ecological communities, causing new species to come into contact or altering existing interactions (Carlson et al., 2022; Moritz et al., 2008). While climate‐driven changes in trophic interactions have received considerable attention (Pecuchet et al., 2020), interactions between hybridizing species under climate change are less well studied.

Hybrid zones, or regions where interbreeding occurs between two genetically distinct groups, are natural experiments that provide insight into evolutionary and ecological processes ranging from speciation and selection on genetic diversity to the mechanisms that maintain species' boundaries. These zones can be broad, such as in *Limenitis arthemis* butterfly subspecies (Ries & Mullen, 2008), or extremely narrow, such as in Western Australian frog species (*Ranidella* genus) (Bull, 1979). They can also be persistent, such as the suture zone across the Great Plains affecting numerous avian taxa (Swenson, 2006), or transient, such as contact zones between invading species like the rusty crawfish (*Orconectes rusticus*) and congeners (Perry et al., 2001). The complex interplay of factors that govern the dynamics of hybrid zones are likely to be highly sensitive to climate change; for example, climate change may alter climatological barriers to gene flow causing new combinations of species to come into contact and alter the strength of selection against hybrids. This sensitivity makes hybrid zones useful in climate change research; they are well‐suited for investigations of the abiotic and biotic selective factors that delimit species' ranges and govern range shifts (Taylor et al., 2015).

Species distribution models (SDMs) are a widely used tool for predicting how species' ranges will change with climate (Hijmans & Graham, 2006), but their use for studying the dynamics of hybrid zones (Swenson, 2006) and forecasting the potential impact of climate change on them is less explored (but see Engler et al., 2013; Guo et al., 2021; Hightower et al., 2023; McQuillan & Rice, 2015). By examining the existing and projected future ranges of parental species using SDMs, researchers can begin to evaluate how climate change may affect hybridization and introgression. While a number of important limitations remain in correlative species distribution modeling and projection of distributions into the future (e.g., Elith et al., 2010; Wiens et al., 2009), they remain an important tool to characterize the range of potential futures facing ecosystems under climate change.

**FIGURE 1:**
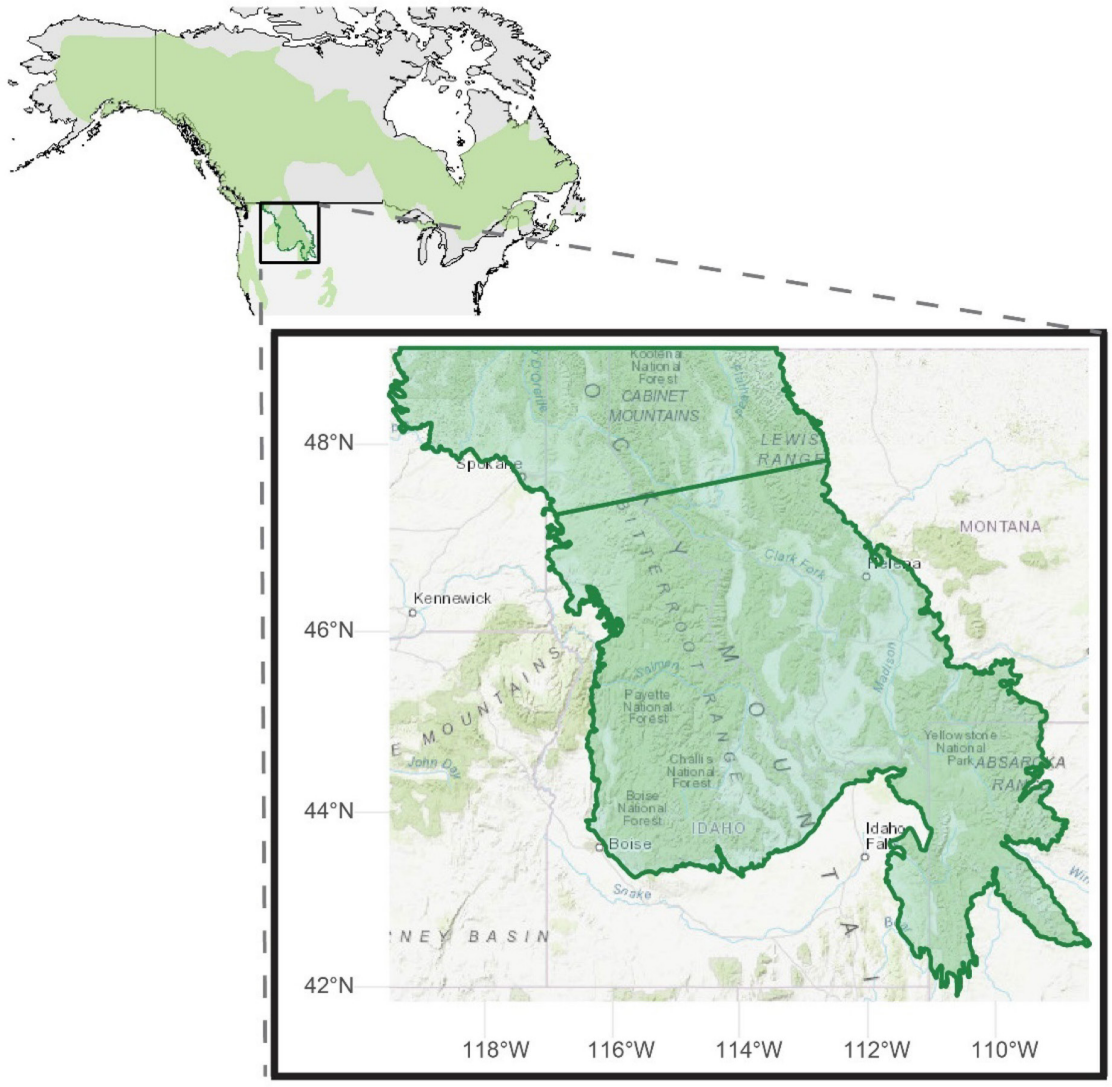
Range of American and Pacific marten (combined) with inset of the study region in the Northern Rockies. The horizontal line dividing the study region is the approximate latitudinal divide (46.78°N) between the current range of American marten (north) and Pacific marten (south) based on genetic detections reported in this study. This divide was used to inform analyses (described in text). Data for combined distribution of American and Pacific marten courtesy of the IUCN.

This study uses a maximum entropy (MaxEnt) modeling approach to characterize the present‐day and potential future distributions of two marten species that come into contact and hybridize in the Northern Rockies. American marten (*Martes americana*) and Pacific marten (*Martes caurina*) (Merriam, 1890) are genetically (Colella et al., 2021; Small et al., 2003; Stone et al., 2002) and morphologically (Colella et al., 2018) distinct species that inhabit forested landscapes of North America. American marten are typically found in more northern locations and Pacific marten are found on the Pacific Coast and in southern locations in the Rocky Mountains (Figure 1). Hybridization of the two species has been described in two contact zones—one in Kuiu Island in southeastern Alaska and the other in Montana (Colella et al., 2019; Dawson et al., 2017; Lucid et al., 2020; Small et al., 2003; Stone et al., 2002; Wright, 1953). Work by Colella et al. (2019) indicates that hybrid offspring are typically the result of male Pacific marten and female American marten interbreeding; they suggest that this bias could be driven by genetic incompatibilities of the two distantly related species (Carr & Hicks, 1997; Colella et al., 2018; Dawson & Cook, 2012; Schwartz et al., 2020). Climate change is predicted to negatively affect marten in North America (Lawler et al., 2012), although comparative studies on climate impacts between the two species have not been done. Specific drivers expected to impact marten include changes in winter snowpack (Lawler et al., 2012), and associated changes in dispersal corridors and genetic connectivity (Wasserman et al., 2013). Marten forage in subnivean spaces and can readily travel across snow‐covered landscapes due to their low foot loading (Raine, 1983). Climate change is also expected to impact marten species by altering competitive interactions between marten and fisher (*Pekania pennanti*) in regions of sympatry (Pauli et al., 2022; Zielinski et al., 2017), potentially through changes in both species' distributions as winter snowpack changes. In characterizing the current and predicted future distributions of both marten species in the Northern Rockies contact zone, we also identify potential impacts to hybrid zone dynamics in this system.

## 2 MATERIALS AND METHODS

### 2.1 Sample collection and genetic analysis

We used a genetic dataset to identify locations currently occupied by American and Pacific marten in the Northern Rocky Mountains. Between 2006 and 2021, hair samples were collected across 56 different research efforts in Northern Idaho, Western Montana, Northeastern Washington, and Northwest Wyoming. Most samples were collected opportunistically during monitoring efforts targeting other mesocarnivores (e.g., wolverine (*Gulo gulo*), lynx (*Lynx canadensis*), fisher). These efforts employed bait stations and wire brushes to lure animals and collect hair samples non‐invasively, and sampling design was such that data could not be analyzed within a presence–absence framework. All fieldwork was conducted with relevant permissions. We focused our analysis on samples collected from this region because information about the site of collection was available at a high spatial resolution and high‐quality DNA extracts were available for genetic analyses to confirm species identities.

We extracted DNA from hair samples with a QIAGEN Dneasy Blood and Tissue kit (Qiagen, Valencia, CA, USA) using protocol modifications for hair described in Schwartz et al. (2020). Extracted DNA was amplified for both mitochondrial DNA (all samples) and 15 microsatellite loci (all samples except those from Northwest Wyoming). Briefly, to determine mitotype (Schwartz et al., 2020), we amplified 389 base pairs (bp) of 16srRNA using primers 16sL 5′‐TTAAACGGCCGCGGTATCC‐3′ and 16sR 5′‐GAATTACGCTGTTATCCCT‐3′. The final 30‐μL reaction volume contained 3‐μL stock DNA extract, 1× reaction buffer (Applied Biosystems, MA, USA), 2.5 mM MgCl2, 200 μM each dNTP, 1 μM each primer, and 1 U Taq polymerase (Applied Biosystems, MA, USA). The PCR program thermal profile was 94°C/5 min, (94°C/1 min, 55°C/1 min, 72°C/1 min 30 s) × 34 cycles, 72°C/5 min. We evaluated quality and quantity of template DNA with 1.6% agarose gel electrophoresis, and purified PCR products using ExoSap‐IT (Affymetrix‐USB Corporation, OH, USA) according to manufacturer's instructions. We sequenced products at Eurofins Genomics (Louisville, KY) using standard Sanger sequencing protocols and used Sequencher (Gene Codes Corps. MI) to compare data to reference sequences.

Second, we genotyped samples at 15 microsatellite loci used in previous studies of mustelids (*Ma1*, *Ma3*, *Ma18*, *Ma19*, *Ggu234*, *Lut604*, *Mp197*, *Mp55*, *Mp85*, *Mp227*, *Ma8*, *Ggu216*, *Mer041*, *Ma2*, *Gg3*) (Dallas & Piertney, 1998; Davis & Strobeck, 1998; Duffy et al., 1998; Fleming et al., 1999; Jordan et al., 2007). The final 10‐μL reaction volume contained 2.5 μL of stock DNA extract, 1× reaction buffer (Applied Biosystems, MA, USA), 2.5 mM MgCl_2_, 200 μM of each dNTP, 1 μM reverse primer, 1 μM dye‐labeled forward primer, 1.5 mg/mL BSA, and 1 U Taq Gold polymerase (Applied Biosystems, MA, USA). The PCR thermal profile was 94°C/5 min ([94°C/1 min, 54°C/1 min, 72°C/30s] × 36 cycles) and we visualized products on a LI‐COR DNA analyzer (LI‐COR Biotechnology, NE, USA). We amplified each sample twice at each locus to screen for allele dropout, stutter artifacts, and false alleles (Dewoody et al., 2006). If a locus did not amplify in both replicates, or if the assigned genotypes differed across replicates, the sample was genotyped one more time in duplicate. If a genotype was confirmed in the second analysis, we retained it; if it failed again, we assigned a missing score to that locus. We removed any sample that failed at more than 30% of loci from downstream analyses. All remaining genotypes were screened using DROPOUT 2.3 to detect and correct genotyping error (McKelvey & Schwartz, 2005; Schwartz et al., 2006) and also in GENALEX v. 6.5 (Peakall & Smouse, 2006) to visualize allele distributions at each locus and identify outlier alleles to re‐amplify.

We used 15 microsatellite loci to assign each sample to a specific individual with an estimated probability of identity (PI; Paetkau & Strobeck, 1994) and probability that siblings are identical (PIsib; Evett & Weir, 1998), which were 6.95 × 10^−16^ and 1.72 × 10^−6^, respectively. We randomly selected one record per individual to build species distribution models. We used 12 of the loci (excluding *Ma3*, *Ma18*, and *Ma19* which were only available for a geographically non‐representative subset of samples) to identify potential hybrids and individuals with mixed ancestry using program STRUCTURE version 2.3.4 (Porras‐Hurtado et al., 2013; Pritchard et al., 2000). We used these classifications to do a basic qualitative evaluation of where admixed individuals were detected relative to the two parental species, and exclude admixed individuals from distribution models. We analyzed microsatellite data in STRUCTURE with an admixture model with *K* = 2 (reflecting the two parental species), which was supported by an analysis comparing models with *K* = 1–10. We ran the analysis without priors and with 10,000 iterations for burn‐in and 10,000 iterations for sampling. We classified individuals with a proportion of ancestry >0.95 to the corresponding parental cluster. Any individual that could not be assigned to a parental cluster with this threshold was classified as having mixed ancestry. Additionally, any individual whose mitochondrial haplotype did not match their species assignment in the STRUCTURE analysis was also classified admixed. Our STRUCTURE results aligned closely with a supplementary analysis of hybridization using a more restricted number of microsatellite loci conducted in NewHybrids (Anderson & Thompson, 2002), which we ran to ensure that our ancestry assignment was not highly sensitive to method (Appendix S1, Table S1, Figures S1 and S2). These methods will identify individuals that are the product of even distant backcrosses, so we have high confidence in our assignment of individuals to parental classes. While future efforts could model hybrid distribution explicitly, the difficulty of assigning specific hybrid classes (F1, F2, backcrosses) confidently with the number of markers used made this beyond the scope of the current study (Appendix S1).

### 2.2 Species distribution modeling and future projection

We modeled the current distributions of both parental species, excluding admixed individuals, in MaxEnt 3.4.3 (Phillips et al., 2006) using the R package *dismo* 1.3–14 (Hijmans et al., 2022) with default settings. Species occurrence data came from the genotyped samples from Idaho, Washington, and Montana (genotyping and filtering described above) and Northwest Wyoming. Samples from Wyoming were assumed to be from Pacific marten as no American marten have been detected in this region. Samples from Wyoming were thinned by randomly selecting only one record from any cell in the study's sampling grid to prevent against pseudoreplication. We did not conduct additional spatial thinning of our presence data because a growing body of literature has suggested that spatial thinning may not improve model performance and may be especially inappropriate for smaller datasets (Gaul et al., 2020; Lamboley & Fourcade, 2024; Ten Caten & Dallas, 2023). We used existing literature on the two species (Baldwin & Bender, 2008; Wasserman et al., 2013; Zielinski et al., 2017) to generate a list of climate, vegetation, hydrological, and topographical variables expected to explain their distribution (summarized in Table 1). We used recent (2020 onwards) vegetation and disturbance products because 70% of our data was collected from 2015 onwards, and because only four individuals (all Pacific marten) used an area before it was affected by fire, meaning that the negative impact of forest disturbance on the alignment of recent vegetation products and conditions at the time of genetic sampling should be minor. For modeling, we reprojected all layers to Conus Albers (epsg: 5070) and resampled them to a 90‐m resolution. We tested for correlations between variables and excluded one if it was highly correlated (>0.7) with another (Dormann et al., 2013). Even though MaxEnt is capable of handling collinear variables with relatively little impact on model performance (Feng et al., 2019); in general, simple models are preferred in applications where they will be transferred across space or time (Merow et al., 2013). When eliminating correlated variables, we preferentially retained those we considered more proximal to defining marten range (e.g., precipitation as snow).

**TABLE 1:** List of covariates considered for inclusion in MaxEnt species distribution models for American and Pacific marten.

Covariate	Layer processing	Source
**Climate**		
Precipitation as snow (normals 1991–2020)	–	Climate WNA (~800 m)
Summer heat to moisture index (normals 1991–2020)	–	Climate WNA (~800 m)
**Topography**		
Elevation	–	NASA SRTM GL3 DEM downloaded from Open Topography (90 m)
Slope	Calculated from elevation with 8 nearest neighbors	NASA SRTM GL3 DEM downloaded from Open Topography (90 m)
Topographic Position Index – 2000 m	Calculated from elevation (scale = 23 with a rectangular window)	NASA SRTM GL3 DEM downloaded from Open Topography (90 m)
**Vegetation**		
Distance to forest edge	Distance from any pixel classified as EVT_LF = “Tree”	LANDFIRE EVT (30 m)
Forest Height	–	LANDFIRE EVH (30 m)
Lodgepole pine	Vegetation class 7050 converted to proportion within 1 km moving window.	LANDFIRE EVT (30 m)
Mixed conifer	Vegetation classes 7045, 7046, and 7166 converted to proportion within 1 km moving window.	LANDFIRE EVT (30 m)
Subalpine Spruce‐fir	Vegetation classes 7055 and 7056 converted to proportion within 1 km moving window.	LANDFIRE EVT (30 m)
Mesic Forest	Veg classes 7047 and 7056 converted to proportion within 1 km moving window.	LANDFIRE EVT (30 m)
Dry Forest	Vegetation classes 7045, 7053, 7055, and 7166 converted to proportion within 1 km moving window.	LANDFIRE EVT (30 m)
Riparian	Vegetation classes 9022 and 9019 converted to proportion within 1 km moving window.	LANDFIRE EVT (30 m)
**Other**		
Distance from water	Distance from any pixel classified as water in “swnet” raster product.	NHD HR Plus (10 m)
Burns	Any burns since 1984 converted to a proportion within a 1 km moving window.	MTBS 2022 Burned Areas Boundaries (30 m)

*Note*: Only climate and topographical variables were included in models used to predict future marten distribution. All vegetation layer processing was done in the R package SpatialEco. Mean annual precipitation, mean summer precipitation, precipitation as snow in the spring, mean coldest month temperature, and mean warmest month temperature were also considered as covariates, but were excluded from the final model because they were highly correlated with other variables.

We defined the study region as the minimum set of EPA Level 3 ecoregions (Northern Rockies, Canadian Rockies, Idaho Batholith, and Middle Rockies; Omernik & Griffith, 2014) that encompassed all marten locations in our dataset. This study region corresponds roughly to the area inhabited by the historic *Martes vulpina* and *abientinoides* subspecies, but excludes the range of the historic *origenes* subspecies further south in the Rocky Mountains (Schwartz et al., 2020). Because our primary purpose was to compare the distribution of the two species across a broad region of potential hybridization in the Northern Rockies, we built models using background samples from the full study region. To test the sensitivity of our findings to this decision, we also built models with background sampling specific to American marten in the northern portion of our study region and Pacific marten in the southern portion of our study region (Appendix S1). Since sub‐regional species‐specific background sampling limits the transferability of findings to the full study region and constrains our ability to compare across species, we consider these results secondary. We generated 10,000 background samples for pseudo‐absences in all models (Phillips & Dudík, 2008). We did not use a mask to exclude areas with water from background sampling because most surveys were conducted in winter and at least one marten was detected on top of a frozen lake. We did not explicitly model sampling bias because it was heterogenous across survey efforts and because background‐correction methods like occurrence‐weighted background sampling exhibit variable performance (Baker et al., 2024; Fourcade et al., 2014) and are highly sensitive to decisions like sampling radius (Baker et al., 2024).

We used the R package blockCV version 3.1‐3 (Valavi et al., 2019) to explore spatial autocorrelation of covariates within our study area and determined that several showed spatial autocorrelation that extended beyond the study area's extent. Based on this, we used k‐fold cross‐validation to build and test models with 90% of data randomly assigned to training and 10% to testing. As such, it is possible that our model's performance is overestimated, as has been documented in the literature for some datasets (Roberts et al., 2017). We assessed model performance using the area under the receiver operating curve (AUC) for testing data (Phillips et al., 2006) and the Continuous Boyce Index (CBI) (Hirzel et al., 2006). The AUC can take values between 0 and 1 (although for presence‐only MaxEnt models, the maximum value may be less than 1, see Phillips et al., 2006) with 0.5 indicating model performance equivalent to a null model. CBI can range between −1 and 1, with positive values indicating predicted distribution where the species is present, zero indicating performance no better than a null model, and negative values indicating predicted distribution where the species is not present. We report these measures as averages (± SD) for all 10 model replicates.

After evaluating model performance with k‐fold cross‐validation, we ran one final model using all data to generate predictive distributional maps for each species. We used the maximum training sum of sensitivity and specificity (Liu et al., 2016), averaged across the 10 cross‐validation runs, to distinguish habitat from non‐habitat and show habitat suitability values above this threshold as continuous values on a complementary log–log (cloglog) scale in figures whenever possible (Phillips et al., 2017). We used this approach so that we could identify areas as suitable for individual marten species, both, or neither while retaining the additional information about habitat suitability above this threshold that continuous data provide. We use permutation importance, as calculated by MaxEnt, to evaluate the relative importance of variables in the model because this metric is insensitive to the order in which variables are added to the model (Phillips, 2017).

Finally, we also generated a present‐day distribution model for each species across the full study region using only climatological and topographic variables so that we could predict how both species' distributions may change under different climate change scenarios (Table 1). While our study site does not encompass the entire realized niche of both species, it is a biologically relevant portion of their niches for climate projection because most American marten habitat excluded from this analysis is in more northern climates and the Northern Rockies Pacific marten population has limited opportunities for gene flow with other Pacific marten populations. We used this present‐day distribution model output, in combination with climate projections from an ensemble of eight Global Circulation models (ACCESS ESM 1.5, CanESM5, CNRM‐ESM 2–1, EC‐Earth3, GFDL‐ESM4, GISS E2‐1‐G, MIROC6, MPI ESM1.2‐HR) for the years 2041–2070 (Wang et al., 2012) to predict where marten habitat will be in the years 2041–2070. We predicted future distributions for each species under the low (SSP126), medium (SSP370), and high (SSP540) emissions scenarios to examine a range of potential futures. We used a threshold based on the maximum sum of sensitivity and specificity to make a coarse assignment of habitat versus non‐habitat so that we could estimate how much marten habitat extent might change under different emissions scenarios and identify areas of our study sites where habitat was likely to be lost, gained, or stable. We quantified collinearity shift between our present and future projection (Dormann et al., 2013; Feng et al., 2019), and found that it never exceeded 0.03, indicating that collinearity shift over time may have little impact on model predictions. We also calculated multivariate environmental similarity surfaces (Elith et al., 2010) for both species to estimate what proportion of habitat estimated for the present day was projected to experience climatic conditions exceeding that of the training data under the SSP126 and SSP540 scenarios. Models reported in the text were run with “clamping” as implemented by MaxEnt for non‐analog environments; however, we also ran models without clamping to see the impact this had on prediction (Appendix S1).

We used Schoener's D (Warren et al., 2008) to compare habitat suitability predictions across different models. To evaluate similarity of predicted niches for the two species, we compared predicted habitat suitability for each across the full study region. To evaluate the impact of modeling decisions on predicted niches, we also compared predictions within species from global models versus climate–topography models.

## 3 RESULTS

Our final dataset included 62 American marten and 219 Pacific marten. We also identified an additional 79 individuals as having mixed ancestry. Forty‐five of these 79 individuals could not be assigned to a parental cluster using STRUCTURE and 34 had a mismatch between mitochondrial haplotype and assignment of parental cluster, with most having an American marten mitotype but assigning to Pacific marten in STRUCTURE. Most admixed individuals could not be assigned to specific hybrid classes (Appendix S1).

**FIGURE 2:**
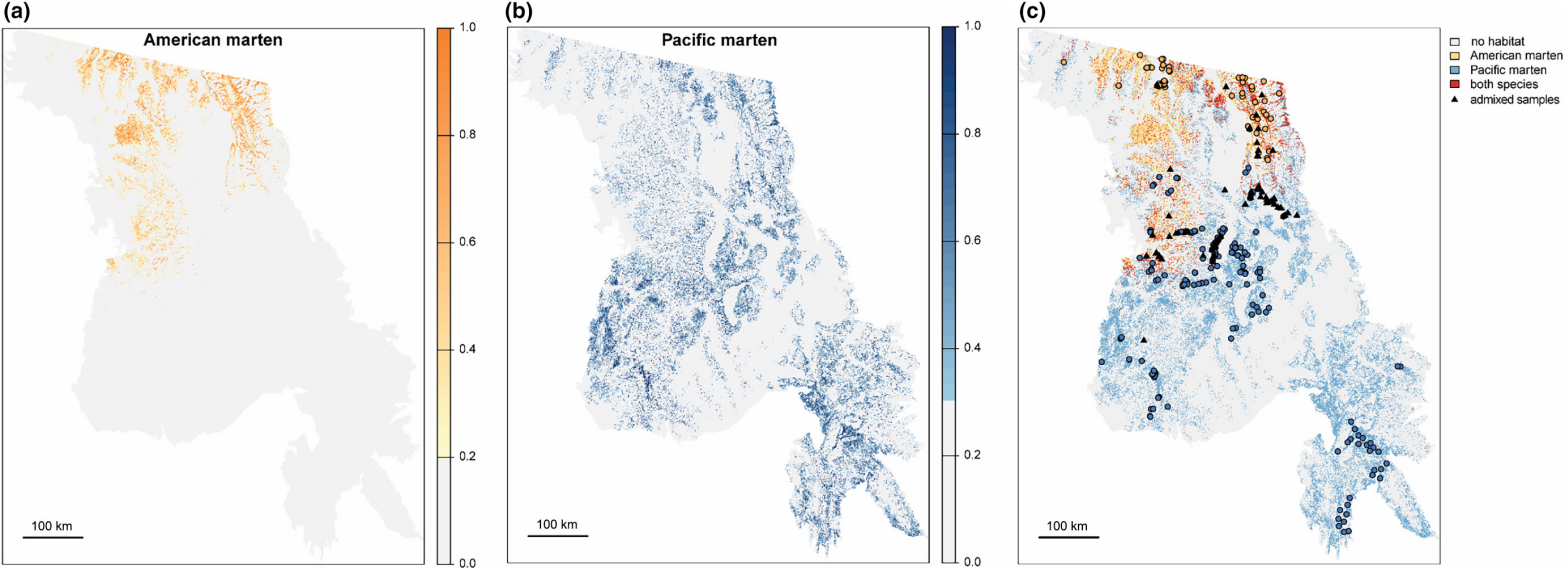
Species distribution models for American marten (a) and Pacific marten (b) built using MaxEnt. Panel (c) compares habitat suitability between the two species and cross‐references modeled habitat with known samples. Gold indicates samples and predicted habitat for American marten, blue indicates samples and predicted habitat for Pacific marten, and red indicates predicted habitat for both species. Black triangles represent samples from admixed individuals.

Distribution models with all covariates performed well under k‐fold cross‐validation. Evaluation criteria exceeded 0.8 for all cases: AUC test (American marten mean = 0.96, SD = 0.02; Pacific marten mean = 0.85, SD = 0.05) and CBI (American marten mean = 0.85, SD = 0.13; Pacific marten mean = 0.82, SD = 0.09). The discriminatory success of models at the threshold based on the maximum sum of sensitivity and specificity was high with the American marten model classifying 94% of actual presence locations as being on suitable habitat and Pacific marten model classifying 84% of actual presence locations as being on suitable habitat. The present‐day distribution models built only with topographic and climate variables performed adequately for both species, although performance declined for American marten models relative to present‐day models that included vegetation covariates: AUC test (American marten mean = 0.96, SD = 0.02; Pacific marten mean = 0.83, SD = 0.03) and CBI (American marten mean = 0.70, SD = 0.37; Pacific marten mean = 0.88, SD = 0.06). The discriminatory success of the climate–topography models was lower than the global models at threshold, with 89% of American marten presence locations and 79% of Pacific marten locations being located on suitable habitat. In general, American marten habitat was predicted in the northern third of our study region and Pacific marten habitat was predicted throughout the study region, but more concentrated in the south (Figure 2).

Habitat for the two species diverged, but the magnitude of divergence varied spatially. Schoener's D comparing predicted American and Pacific marten distribution was 0.267 (global model)/0.31 (climate–topography model). Only 2.9% (global model)/4% (climate–topography model) of the total study area was identified as being suitable for both species (Figure 2). Within the northern portion of the study region, predicted niche overlap for the two species was 0.502, and habitat identified as suitable for both species covered about 7.5% of the landscape. In contrast, within the southern portion of the study region, predicted niche overlap was 0.213, and 1.4% of lands were suitable for both. Samples coming from animals with mixed ancestry were found in locations near predicted habitat for both species (i.e., midlatitudes and along the northeastern flank of the study region) (Figure 2c). However, on small (30 m) spatial scales, most samples from individuals with mixed ancestry were taken from points identified as Pacific marten habitat (*n* = 33), with two in locations identified as suitable for American marten, and 19 in locations identified as suitable for both species. An additional 25 individuals were located in habitat that did not exceed the suitability threshold for either species. These findings should be interpreted with caution given the spatial scale of prediction (30 m) and small‐scale variation in GPS precision during the 20 years of this study. The niche overlap predicted by models with all covariates versus models with only climate and topography variables was 0.76 for American marten and 0.74 for Pacific marten (Figure S4).

Elevation (mean = 54.2%, SD = 2.6%) and summer heat moisture index (mean = 30.1%, SD = 1.69%) (Table 2) were influential variables in the global American marten distribution model and these covariates performed similarly (elevation mean = 53.3%, SD = 5.4%; summer heat moisture index mean = 43.6%, SD = 7.3%) in the climate–topography. Marginal response curves (generated with other variables held at median values) show that American marten were expected at intermediate elevations (~1200 m) and in locations with low summer heat moisture index values (less than 50), which correspond to summer conditions of low temperatures and high precipitation (Figure S6).

**TABLE 2:** Average variable permutation importance scores (±SD) calculated from MaxEnt output for 10 model replicates, for each model formulation across the entire study region.

	Variable	Variable importance	SD
Climate–Topography Model: American marten	Elevation	53.2581	5.383432
	Summer Heat Moisture Index	43.5723	7.302995
	Precipitation as Snow	2.5642	1.900029
	TPI	0.3929	0.185462
	Slope	0.2124	0.111486
Climate–Topography Model: Pacific marten	Precipitation as Snow	38.903	2.197
	Slope	35.036	2.148
	TPI	19.261	1.519
	Elevation	3.768	0.894
	Summer Heat Moisture Index	3.033	1.182
Global Model: American marten	Elevation	54.2272	2.59759
	Summer Heat Moisture Index	30.1222	1.69161
	Spruce Fir Forest	5.3013	1.083717
	Precipitation as Snow	2.6181	0.639272
	Forest Height	2.0805	0.841286
	Wet Forest	1.5661	0.551842
	Dry Forest	1.1261	0.436481
	Distance to water	0.9446	0.597347
	Riparian	0.6553	0.50072
	Topographic Position Index	0.4487	0.36781
	Mixed Conifer Forest	0.2856	0.406415
	Slope	0.2276	0.185078
	Distance to Forest Edge	0.1783	0.185147
	Lodgepole Forest	0.1454	0.336539
	Area Burned	0.073	0.058544
Global Model: Pacific marten	Slope	19.6154	2.8196
	Mixed Conifer Forest	16.0865	3.7253
	Distance to Forest Edge	9.4538	4.8773
	Topographic Position Index	8.3082	1.7897
	Lodgepole Forest	6.7911	1.4246
	Summer Heat Moisture Index	5.9816	1.0860
	Elevation	5.8646	1.6147
	Wet Forest	5.8319	1.4005
	Precipitation as Snow	5.4137	2.6178
	Forest Height	3.9576	1.5192
	Spruce Fir Forest	3.6319	0.9387
	Area Burned	2.8741	0.9520
	Dry Forest	2.6439	0.8055
	Distance to water	2.0631	0.7411
	Riparian	1.4824	0.7166

*Note*: The leftmost column identifies the model for which results are presented.

The most important variables for predicting Pacific marten distribution in the global model were slope (mean = 19.6%, SD = 2.8%), proportion of mixed conifer forest (mean = 16.1%, SD = 3.7%), and distance to forest edge (mean = 9.5%, SD = 4.9%) (Table 2). Slope remained important in the climate–topography model (mean = 35.0%, SD = 2.1%), and precipitation as snow rose in importance (mean = 38.9%, SD = 2.2%) in the absence of vegetation covariates. Marginal response curves for the global model showed that habitat was predicted in areas with no or slight slopes (<20°) and within the forest or immediately adjacent to forest edges (<1 km) (Figure S7). Pacific marten habitat suitability was higher in areas with low proportions of mixed conifer. In the model built using climate and topographic variables, marginal response curves for precipitation as snow showed that habitat suitability was high when precipitation as snow exceeded 250 mm annually.

**FIGURE 3:**
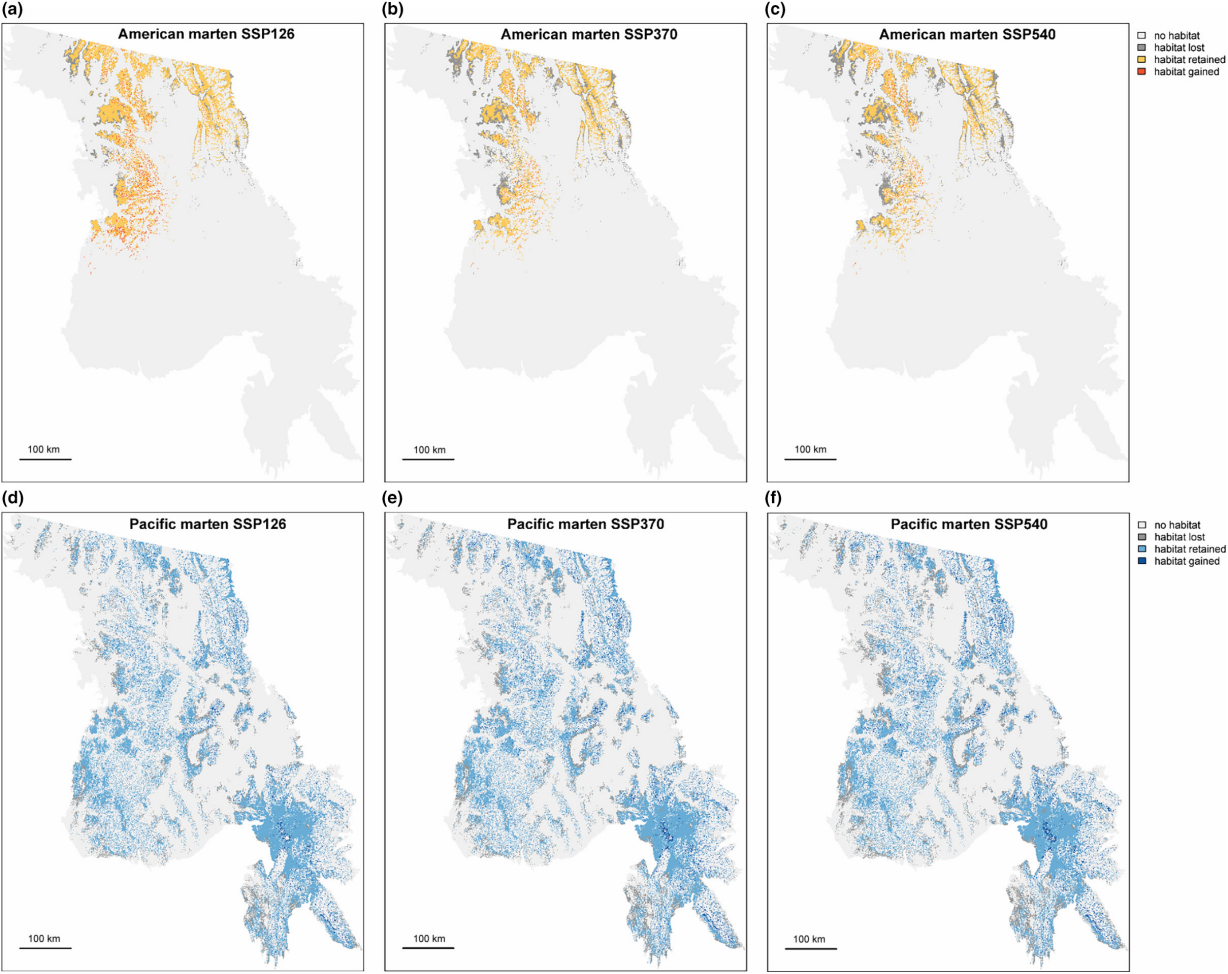
Maps depicting predicted marten distribution for the period 2041–2070 by species (a–c: American marten, d, e: Pacific marten) under low, medium, and high emissions scenarios. Color coding represents habitat lost, retained, or gained relative to the present‐day species distribution. Maps showing continuous output are provided in Figure S5.

**FIGURE 4:**
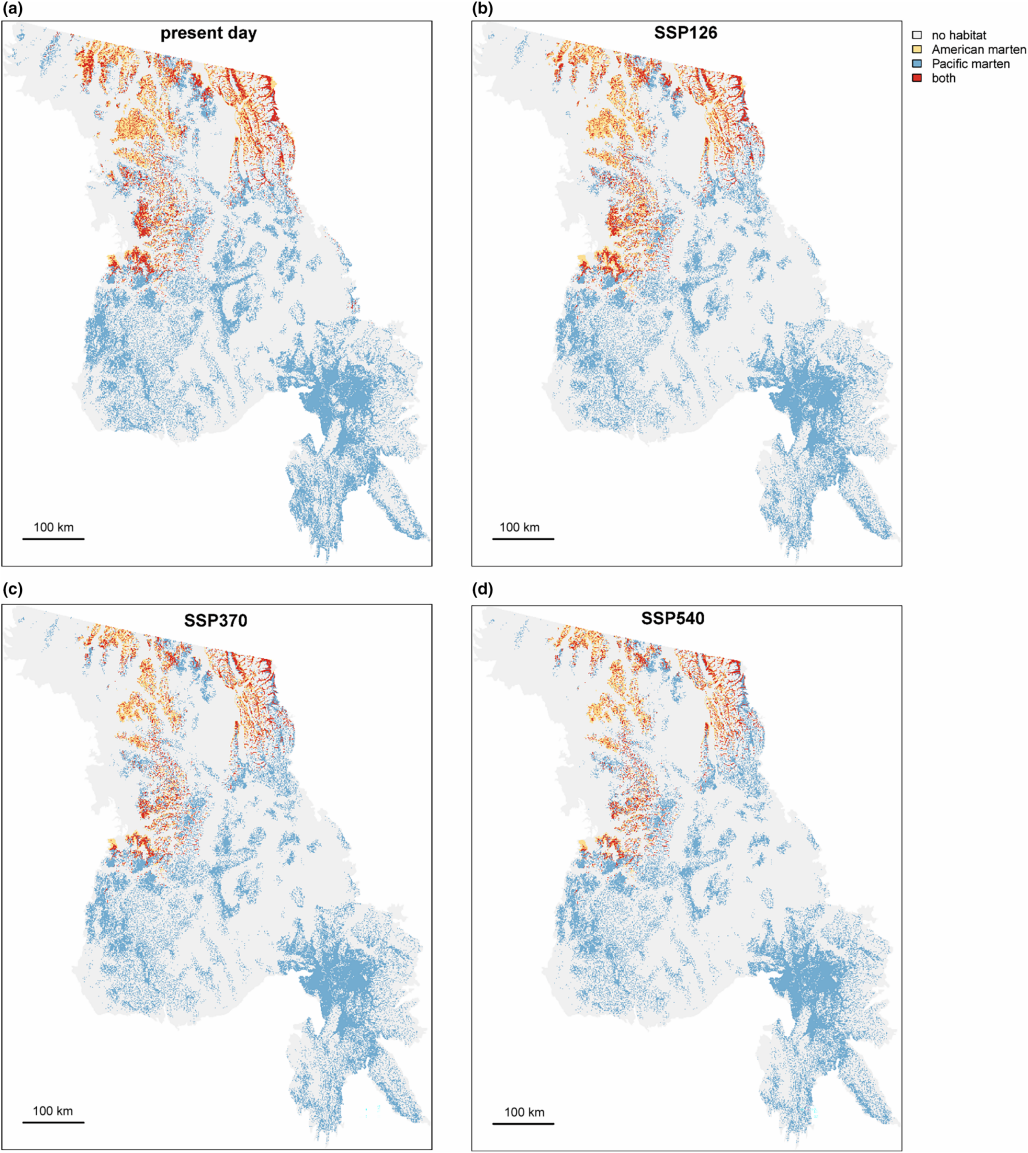
Overlap of suitable habitat for American and Pacific marten as predicted by a model containing only climate and topographic variables. Panel (a, top left) shows predictions for the present day, and panels (b–d) show predictions under SSP126, SSP370, and SSP540 emissions scenarios for the period 2041–2070.

The amount of suitable habitat predicted for both marten species decreased under all emissions scenarios (Figure 3). By the mid‐21st century, declines of 8% (SSP126), 35% (SSP370), and 40% (SSP540) were predicted for American marten. Declines in suitable habitat predicted for Pacific marten were smaller at 14% (SSP126), 15% (SSP370), and 19% (SSP540), respectively. For American marten, habitat was lost at the lower elevations, with losses outstripping habitat gains at higher elevations. The small amounts of habitat currently predicted for American marten in the Big Belt Mountains, Scapegoat Wilderness, and southern portion of the Bob Marshall Wilderness were mostly lost, or completely lost, under all emissions scenarios (Figure 3). Pacific marten also lost predicted suitable habitat at lower elevations and gained some predicted suitable habitat at higher elevations under all emissions scenarios (Figure 3). The total amount of the study area that was identified as suitable for both species declined relative to the present day from ~4% of the study area to ~2.4% in the SSP540 emissions scenario (Figure 4). Predictions for both species, and overlap between species, were affected by model assumptions about how species respond to climate conditions that exceed those of presence training data: conditions were projected to extend beyond the training data range for 10% (SSP126)‐ 58% (SSP540) of present‐day American marten habitat. For Pacific marten, 3% (SSP126)–13% (SSP540) of present‐day habitat was projected to experience novel conditions. Projected habitat losses were similar in models run without clamping, but differed in absolute amount of habitat predicted for each time period and climate scenario (Appendix S1).

## 4 DISCUSSION

In our study area, American marten and Pacific marten have distinct distributions. While the Northern Rockies is a zone of contact and hybridization between the two species, only a small portion of our study region was identified as suitable for both. Suitable American marten habitat and non‐invasive genetic detections for this species were located exclusively in the northern half of our study region. Some Pacific marten habitat was predicted throughout the latitudinal range of our study region; however, non‐invasive genetic detections were located only in the most southerly 2/3 of the study region.

These findings shed light on factors influencing the realized range for both species. The absence of Pacific marten from more northerly latitudes, despite availability of apparently suitable habitat, could be evidence that interactions with American marten are excluding Pacific marten from the north. This is somewhat unexpected as abiotic, not biotic, factors are expected to be more important for defining species' northern range limits (Paquette & Hargreaves, 2021). Additional work is required to assess whether intra‐specific interactions cause Pacific marten to leave a portion of suitable habitat unoccupied, or whether other factors, such as the long‐term evolutionary history of the two species and expansion since the last glacial maximum explain this pattern. For American marten, genetic detections align closely with predicted habitat, being concentrated in the northern portion of the study region. The notable exception is the Coeur d'Alene National Forest, which has high habitat suitability, but lacks genetic detections potentially due to low survey effort in this region relative to other portions of the study area. Of note, one of the previously published systematic surveys of this region had low number of marten detections here (Lucid et al., 2020). This is an area where fisher habitat suitability is high (Olson et al., 2014), and it is possible that competition from this species could add additional complexities to marten dynamics in this region (Zielinski et al., 2017).

There were some surprises in the influential variables that predicted distribution for the two species. Precipitation as snow did not influence distribution predictions strongly in models built with all covariates, but was highly influential in models for Pacific marten built with only climate and topographic variables (but not American marten). A review of density plots revealed that American marten are present in areas with at least a minimum amount of snow, but the difference between the amount of precipitation as snow at presence locations (median presence: 369 mm, range: 181–809 mm), versus pseudo‐absence locations sampled from across the full study region (median absence: 271 mm, range: 19–1668 mm) was not large. Precipitation as snow rose in importance when American marten distribution was modeled with background sampling only in the northern portion of the study site (Appendix S1), and the influence of other covariates in the model shifted. The difference in performance of precipitation as snow across model formulations highlights the challenges of inferring the biological significance of variables from statistical importance in correlative species distribution models. The biological significance of snow to marten species is well documented, despite the sensitivity of precipitation as snow's performance to modeling decisions. The influence of numerous statistical factors, such as study region, spatial resolution of covariates, and other modeling decisions, on the assessment of variable importance in MaxEnt has been examined in recent simulation work (Smith & Santos, 2020) and these factors may explain this result.

While only a small portion of our study area was identified as suitable for both species, mostly along the Highway 12 corridor in Western Montana and in the mountain ranges to the east of Flathead Lake near Glacier National Park and the Bob Marshall Wilderness, we detected individuals with mixed ancestry near these general locations. This study, and at least one other (Colella et al., 2019), indicate that hybridization occurs more often from American marten females breeding with Pacific marten males, although it is not clear whether mate choice (by either sex), competition, dispersal, differential survival of hybrids based on maternal ancestry, or other factors drive this pattern. Our finding of a bias in maternal ancestry of admixed individuals, in combination with our finding that Pacific marten may not be occupying the full suite of habitat identified as suitable for them, suggest that future work investigating the behavioral, physiological, and other factors underpinning species interactions at this hybrid zone is warranted.

Climate change is projected to decrease habitat for both species by the 2041–2070 time period, regardless of emissions scenario. This is concomitant with changes in summer heat moisture index, which increased, and precipitation as snow, which decreased. Effects were particularly dramatic for American marten, which were predicted to lose 35–40% of current habitat in the study region under moderate and high emissions scenarios. While American marten are widely distributed in Canada and Alaska, meaning that they will likely continue to persist outside of our study region, our findings suggest that populations at their southern range periphery in the Northern U.S. Rockies may be under pressure with global climate change. This is consistent with another model, which did not distinguish between the two marten species, which predicted that by the end of the twenty‐first century marten would lose most suitable habitat in the contiguous United States (Lawler et al., 2012). While predicted effects of climate change on habitat suitability for Pacific marten were less acute, they should not be ignored. The impacts of climate change on Pacific marten within this study region are proportionally smaller than those predicted for American marten, but given that the total range of Pacific marten is also much smaller, this represents a larger portion of their range. Additionally, Pacific marten in the Northern Rockies are relatively isolated from other Pacific marten populations on the western coast of North America, there is regional genetic substructure for this species (Schwartz et al., 2020), and they occupy different habitats. Therefore, it may be most appropriate to consider climate impacts on Pacific marten in the Northern Rockies separately from those of other populations.

There are several factors that influence our interpretation of projected distributional changes under climate change. First, climate and topographic variables used to project distributions are an imperfect representation of the suite of abiotic and biotic factors that define habitat. Given that key habitat features (e.g., vegetation) may face considerable lags in turnover as the climate changes (Aitken et al., 2008), this is important for users of these projections to consider. Relatedly, our estimates of current suitable habitat are sensitive to the modeling method (i.e., predictions from global models vs. climate–topography models). Given that models without vegetation variables consistently estimated more habitat than those including vegetation variables (25.0% vs. 19.2% of the study area for Pacific marten and 8.4% vs. 7.7% of the study area for American marten), it is possible that our projections are overestimates of future marten habitat. Second, estimates for both current and future distribution of Pacific marten would shift if the range was modeled using samples from other populations in the Middle Rockies or on the West Coast, although as noted it may be most appropriate to treat these populations separately. The impacts of adding samples from American marten in Canada and Alaska to habitat projections would likely be more minor as we expect that conditions at our study site will come to represent those further south as the climate continues to warm. Our models were run using “clamping” in MaxEnt, which holds model predictions at a constant value reflecting those from the extremes of the training data when predictions are made for conditions exceeding the training data (as opposed to extrapolating relationships to new conditions). While invoking clamping is common practice and the default setting for MaxEnt software, given that we cannot know how species will respond to non‐analog environments without mechanistic investigations, both clamped and unclamped models reflect an untested assumption. One solution is to run models with different clamping rules and explore a range of scenarios—while our estimates of proportionate habitat lost were similar across models with and without clamping, the total area estimated for each scenario differed, especially for American marten. Given that results from our multivariate environmental similarity surface analyses show that present‐day marten habitat will experience novel conditions under both low and high emissions scenarios, investigating how marten may respond to novel conditions is important to understanding the impacts of climate change on this species. It is also important to note that our models cannot account for the influence of inter or intra‐specific interactions on marten ranges, unless they are strongly correlated with the abiotic factors used in models, or the role of phenotypic plasticity (whether adaptive or maladaptive) in influencing realized species' ranges.

While we did not explicitly model habitat suitability for hybrids, shifts in the distribution of parental species could result in shifts in the hybrid zone. Our models revealed a decrease in the total amount of predicted suitable habitat overlap for the two species and habitat overlap was lost along the Highway 12 corridor in the middle portion of the study region under the higher emissions scenarios. This suggests the potential for decreasing interactions between the species with climate change, which could impact hybridization dynamics as well as the ranges for each parental species. Other studies have demonstrated shifts in hybrid zones with global climate change (Alexander et al., 2022; Ryan et al., 2018; Taylor et al., 2014), although attributing causes to hybrid zone shifts remains challenging (Buggs, 2007). Future studies could potentially leverage genetic sequencing methods that sample a greater proportion of the genome (i.e., whole genome or reduced representation sequencing) to more finely interrogate the extent and degree to which hybridization is occurring in this zone and how it is affected by climate change.

## 5 CONCLUSION

Marten in the Northern Rockies offer an interesting system to examine how biotic and abiotic factors inform species' ranges and how climate change may alter range dynamics in a hybrid zone via both mechanisms. Our data show that American and Pacific marten in the Northern Rockies occur in distinct locations and may be affected by climate change differently. This could impact the degree of contact between these species in the future. These species‐specific needs and predicted climate change impacts should be considered by managers charged with managing harvest, translocation, and other important decisions for these species.

## AUTHOR CONTRIBUTIONS

**Helen E. Chmura:** Conceptualization (lead); data curation (lead); formal analysis (lead); investigation (lead); methodology (lead); visualization (lead); writing – original draft (lead); writing – review and editing (lead). **Lucretia E. Olson:** Conceptualization (supporting); formal analysis (supporting); methodology (supporting); writing – review and editing (supporting). **Remi Murdoch:** Data curation (supporting); investigation (supporting); methodology (supporting); writing – review and editing (supporting). **Alexandra K. Fraik:** Formal analysis (supporting); methodology (supporting); visualization (supporting); writing – original draft (supporting); writing – review and editing (supporting). **Scott Jackson:** Funding acquisition (equal); investigation (supporting); writing – review and editing (supporting). **Kevin S. McKelvey:** Conceptualization (supporting). **Rex Koenig:** Conceptualization (supporting). **Kristine L. Pilgrim:** Conceptualization (supporting); data curation (supporting); investigation (supporting); methodology (supporting); supervision (supporting); validation (lead); writing – review and editing (supporting). **Nicholas DeCesare:** Writing – review and editing (supporting). **Michael K. Schwartz:** Conceptualization (supporting); formal analysis (supporting); funding acquisition (equal); methodology (supporting); resources (lead); writing – review and editing (supporting).

## FUNDING INFORMATION

Funding for this project was provided by the US Forest Service and the National Genomics Center for Fish and Wildlife Conservation.

## CONFLICT OF INTEREST STATEMENT

The authors declare no conflicts of interest.

## Supporting information


Appendix S1.

## Data Availability

Data are publicly available on Dryad at DOI: 10.5061/dryad.zpc866tgs. Spatial coordinates are provided at a 10‐km resolution because American and Pacific marten are harvested species and hair sampling for this project occurred at monitoring sites for wolverine and Canada lynx, which are protected under the Endangered Species Act.
