# Supplementary material for: Climate change differentially alters distribution of two marten species in a hybrid zone
Source: Ecol Evol. 2024 Aug 19;14(8):e70181. doi: 10.1002/ece3.70181 (PMC11333536; doi:10.1002/ece3.70181)

**Supplementary Materials**

**Supplementary analysis of marten ancestry in NewHybrids and comparison with other methods**

We ran a Bayesian MCMC method in NewHybrids (v1.3) to identify admixed individuals of the two marten species and make sure that our identification of admixed individuals was not sensitive to analytical method. The underlying genetic model for NewHybrids is similar to the model underlying STRUCTURE, however, it assumes more recent genetic admixture among individuals of unknown origins (Anderson and Thompson 2002). The analytical approach in NewHybrids does not require data from pure individuals of either parental species or known allele frequencies of genetic markers for parental species (Anderson and Thomspon 2002; Anderson 2008) and does not assign individuals to a single class. Instead, it computes a posterior probability that sampled individuals belong to multiple different hybrid classes (pure species 0, pure species 1, first generation hybrid, second generation hybrid, backcross to species 0 and backcross to species 1). The posterior probabilities account for uncertainty in model parameters, including uncertainty in the allelic frequency at each locus within each hybrid class. It also allows for assignment of priors to individuals of known origin.

Analyses in NewHybrids can be run with and without priors and we ran our analyses both ways to evaluate whether assignments to different hybrid classes (F1, F2, backcross species 0, backcross species 1) were sensitive to prior specification. Analyses used data from 9 microsatellite loci (*Ma1, Ma2, Ma8, Gg3, Ggu216, Ggu234, Mer041, MP197, Lut604*) for which we had data from samples collected in our study region and pure parental populations in California, Washington, and Oregon (Pacific marten, pure reference species 0 (“z0”), n=426) (Schwartz et al., 2020) and Alaska and New York (American marten, pure reference species 1 (“z1”), n=46). Our first analysis did not include data from reference populations as priors, our second did. Individuals included as our pure references were genotyped to species using the species diagnostic mitochondrial haplotype. Each analysis was run for a burn in period of 10,000 with 500,000 sweeps to allow for model convergence. We then assessed how concordant species identification was between NewHybrids analyses, the STRUCTURE results reported in the main text, and species-specific mitochondrial haplotypes. Individuals were considered pure parental species if STRUCTURE results (admixture model, K=2) showed they had high proportions of ancestry (> 95%) to a single genetic cluster (individuals with <95% proportions of ancestry to both clusters were labeled admixed). Individuals were considered pure parental species in NewHybrids analyses if the cumulative posterior probability of assignment to any combination of hybrid classes was >95%. We also assessed whether individuals had high, consistent, assignments to specific hybrid classes (>95% posterior probability of assignment to a single hybrid class) across the two NewHybrids analyses.

We found significant overlap for conspecific assignment across all methods (Table S1). 249 individuals were consistently assigned as American marten (N = 61) and Pacific marten (N = 188) (Table S1) when the NewHybrids analysis did not include prior information. With the inclusion of prior information in the NewHybrids analysis, 215 individuals were consistently assigned to species with 48 identified as American marten and 167 as Pacific marten (Table S1). There was some concordance in assignment of admixed individuals across STRUCTURE and NewHybrids analyses (n=23 without priors, n=14 with priors; Table S1), which reflects the greater uncertainty in NewHybrids results. Hybrid class assignment was only possible for some individuals in NewHybrids (n=23 with priors, n=14 without priors) as most individuals classified as hybrids had a low posterior probability of assignment (<95%) to a specific hybrid class. This pattern of having high confidence in identifying an individual as admixed, but low confidence in the assignment of an individual to a specific hybrid class, has also been seen in NewHybrids simulations done with a limited number of genetic markers (Anderson 2008).

Since our goal was to use a conservative approach to exclude admixed individuals from species distribution models, we used both STRUCTURE results and mitochondrial haplotype to screen samples for admixture, which resulted in a final assignment of n=62 (American marten), n=219 (Pacific marten) and n=79 (admixed). There were only 4 individuals that STRUCTURE and mitochondrial haplotype assigned to the same parental species and that could not be assigned with confidence in the NewHybrids analysis without priors (American marten n=1, Pacific marten n=3). There were additional individuals that NewHybrids could not confidently assign in the analysis with priors, but because the pure reference populations used in the analysis had unique alleles that were not present in our study region, we have higher confidence in the NewHybrids analysis run without priors. Given this, it is unlikely that our species distribution model results are sensitive to the analytical methods we used to identify admixed individuals and exclude them from distribution modelling.

**Supplementary analysis using species-specific background sampling**

To evaluate the sensitivity of our analysis to background sampling assumptions, we conducted a second set of analyses using “species-specific” background sampling for the model predicting present-day distribution with all covariates. While this scale is less appropriate for the primary question of our study, given the well documented importance of background sampling decisions on species distribution models (reviewed in: Merow et al. 2013), it was an important sensitivity analysis to conduct. To do this analysis, we subdivided our study region into two “species-specific” study regions along 46.78°N, which is the approximate latitudinal mid-point between the northernmost Pacific marten and the southernmost American marten samples in our dataset (Figure 1). While the zone of contact between the two species is proposed to run in a more NW to SE direction (e.g. Colella et al. 2019), instead of a strict North/ South divide, no study to date has delineated this zone at high resolution meaning a more refined rule for subdivision was not available (but see Colella et al. 2019, Lucid et al. 2020 for other studies in the region). Aside from changes to background sampling, all other modelling decisions remained the same as those reported in the main text. Within the northern American marten study region, correlations among elevation, precipitation as snow, and summer heat moisture index all exceeded 0.7, compared to 0.5-0.7 for the full study region and southern Pacific marten study region. Despite this, we retained the same suite of predictors we had used for the models built with background sampling across the full study region to ensure comparability as MaxEnt can handle high levels of collinearity (Feng et al. 2019). Since we planned to conduct analyses looking at the similarity of predictions generated between models with full study region and species-specific background sampling across the full study region, we ran correlation shift tests to see how the relationships between environmental covariates shifted across the different sampling regions, and thus the impact changes in variable correlation structure might have on model transferability(Dormann et al. 2013). Correlation shift tests indicated that the maximum absolute difference in variable correlations between full and restricted ranges were 0.32 (American marten) and 0.13 (Pacific marten) so predictions from the transfer of the American marten-specific background sampling model to the full study region should be treated with caution (Dormann et al. 2013). To compare the impact of background sampling decisions on model predictions, we used Schoener’s D to compare predicted habitat within each species for both full and species-species background sampling.

Performance of models built using species-specific background sampling performed well: AUC test (American marten mean=0.88, SD=0.05; Pacific marten mean= 0.85, SD=0.05) and CBI (American marten mean=0.91, SD=0.07; Pacific marten mean=0.87, SD= 0.06). These performance metrics are comparable to those reported for models built using full study region sampling- with the full study region models slightly out-performing species-specific models for some criteria, and species-specific models performing better under other criteria.

The impact of background sampling method on predicted species distributions was species-specific (Figure S3). While the models with full study region and species-species specific background sampling predicted similar American marten distributions within the smaller American marten study region (D=0.863) they diverged when predicting distribution across the full background region (D=0.669), which may be due to the variable correlation shifts identified previously. Within the American marten study region, the predictions generated using the two background sampling rules agreed on classification of suitable vs. unsuitable habitat 89% of the time (Figure S3). Twenty-three percent of the American marten-specific study region was identified as suitable habitat in one of the two models, with 54% of habitat being identified as suitable by both, 44% of habitat identified by only the full study region model, and 2% identified in the restricted background sampling model.

The most important variables changed moderately when American marten distribution was modeled using background samples from only the northern American marten study region instead of the full study region (Table S2). While summer heat moisture index remained important (mean= 40.7%, SD= 3.00%), elevation decreased in importance dramatically (mean= 6.3%, SD= 2.3%). With the declining importance of elevation, precipitation as snow (mean=10.7%, SD=4.35%) and forest height (mean=10.9%, SD=2.78%) rose in importance (Table S2). Marginal response curves show that habitat was expected in areas that received less than 0.5m of snow a year and that had taller tress (~ 30m). This was not because American marten were not found in regions with more than 0.5m of snow, but because detection rates in those regions did not exceed those of background.

Distributions predicted for Pacific marten were similar for the two background sampling rules whether they were predicting distribution within the Pacific marten specific study region (D=0.870) or across the full background region (D=0.825) (Figure S3). Within the Pacific marten-specific study region, the predictions generated from models using the two background sampling approaches agreed on classification of suitable vs unsuitable habitat 93% of the time (Figure S3). Within that southern region, 23% of lands were identified as suitable habitat in at least one of the two models, with 69% of lands identified as suitable by both, 22% identified as suitable only by the model using full study region background sampling and 8.7% identified as suitable only by the model using background sampling from the southern portion of the study region.

The most important variables also shifted somewhat for Pacific marten depending on background sampling rules (Table S2). Compared to the model with full background sampling, slope (mean=19.5%, SD=2.15%) and distance to forest edge (mean=10.1%, SD=3.15%) performed very similarly. Relative to results from the model built with full study region background sampling, mixed conifer forest declined slightly in importance (mean=9.2%, SD= 2.7%) in the model built with Pacific marten specific background sampling. In contrast, the importance of precipitation as snow (mean= 12.5%, SD=4.33%) and proportion of lodgepole pine forest (mean=11.5%, SD=1.67%) increased in permutation importance (Table S2). Pacific marten habitat was predicted in places with intermediate proportions of lodgepole pine within 1 km (~60%) and with at least 250mm of snow a year.

These findings show that while the relative importance of different variables to distribution models only changed a little bit across the two background sampling paradigms for Pacific marten, they changed moderately for American marten. This seems to be driven by the changes in variable correlations between elevation, precipitation as snow, and summer heat moisture index between the full study region and the northern, American marten specific study region and the relative availability of habitat with particular values for these covariates across the two. As such, it is important for those using these results to think carefully about the difference between the biological and statistical importance of these variables for marten(Smith and Santos 2020).

**Supplementary analysis examining the impact of clamping on model predictions**

To evaluate the impact of MaxEnt’s “clamping” setting on model predictions, we conducted a supplementary analysis that modeled habitat without clamping for the present day and each of the three emissions scenarios for both species. We used model predictions to estimate habitat loss relative to the present day.

For both species, models built without clamping predicted more suitable habitat (in total area) than models with clamping. For American marten, the total area without clamping was 61.9% greater than model predictions with clamping. Clamping affected both the habitat suitability values predicted by models, as well as the threshold value at which sensitivity and specificity was maximized. These factors combined meant that the model without clamping predicted more American marten habitat at higher elevations than the model with clamping. Despite the differences in total habitat estimated, the proportion of habitat lost under emissions scenarios was very similar across modelling assumptions, especially under higher emissions scenarios with predictions of 17.1% (SSP126), 34.7% (SSP370) and 39.1% of habitat lost by the 2041-2070 time period. Total amount of habitat predicted for Pacific marten was more similar across modeling approaches with models without clamping predicting 7.7% more habitat than models without clamping. Proportionate losses relative to the present day were similar regardless of model specification with 15.4% (SSP126), 16.9% (SSP370), and 21.2% (SSP540) of predicted habitat lost by the 2041-2070 time period.

**Supplementary References:**

Anderson EC. 2008. Bayesian inference of species hybrids using multilocus dominant genetic markers. *Phil. Trans. B.* 363:2841-50. doi: 10.1098/rstb.2008.0043.

Anderson E.C., and E.A. Thompson. 2002. A model-based method for identifying species hybrids using multilocus genetic data. *Genetics*. 160(3):1217-29. doi: 10.1093/genetics/160.3.1217.

Colella, J. P., R. E. Wilson, S. L. Talbot, and J. A. Cook. 2019. Implications of introgression for wildlife translocations: the case of North American martens. Conservation Genetics 20:153–166.

Lucid, M., S. Cushman, L. Robinson, A. Kortello, D. Hausleitner, G. Mowat, S. Ehlers, S. Gillespie, L. K. Svancara, J. Sullivan, A. Rankin, and D. Paetkau. 2020. Carnivore contact: A species fracture zone delineated amongst genetically structured North American marten populations (Martes americana and Martes caurina). Frontiers in Genetics 11.

Merow, C., M. J. Smith, and J. A. Silander Jr. 2013. A practical guide to MaxEnt for modeling species’ distributions: what it does, and why inputs and settings matter. Ecography 36:1058–1069

Schwartz, M. K., A. D. Walters, K. L. Pilgrim, K. M. Moriarty, K. M. Slauson, W. J. Zielinski, K. B. Aubry, B. N. Sacks, K. E. Zarn, C. B. Quinn, and M. K. Young. 2020. Pliocene–Early Pleistocene geological events structure Pacific martens *(Martes caurina*). Journal of Heredity. 111:169–181.

Smith, A., and M. Santos. 2020. Testing the ability of species distribution models to infer variable importance. Ecography 43:1801–1813.

Table S1: Concordance across analytical methods in assignment of parental species and admixed individuals. Results shown for STRUCTURE, mitochondrial haplotype and NewHybrids analyses with and without priors.

| No priors | | |  |  |
| --- | --- | --- | --- | --- |
|  | American marten | Pacific marten | >1+ Hybrid Class | Low Probability of Assignment |
| mtDNA | 110 | 223 | NA | NA |
| STRUCTURE | 64 | 225 | 44 | NA |
| NewHybrids | 63 | 221 | 23 | 26 |
| Consensus mtDNA & STRUCTURE | 62 | 193 | NA | NA |
| Consensus mtDNA & NewHybrids | 61 | 190 | NA | NA |
| Consensus STRUCTURE & NewHybrids | 63 | 219 | 23 | NA |
| Consensus across all three methods | 61 | 188 | NA | NA |

| Priors | | |  |  |
| --- | --- | --- | --- | --- |
|  | American marten | Pacific marten | >1+ Hybrid Class | Low Probability of Assignment |
| mtDNA | 110 | 223 | NA | NA |
| STRUCTURE | 64 | 225 | 44 | NA |
| NewHybrids | 52 | 199 | 14 | 68 |
| Consensus mtDNA & STRUCTURE | 62 | 193 | NA | NA |
| Consensus mtDNA & NewHybrids | 51 | 174 | NA | NA |
| Consensus STRUCTURE & NewHybrids | 49 | 190 | 14 | NA |
| Consensus across all three methods | 48 | 167 | NA | NA |

**Table S2:** Average variable permutation importance scores (± SD) calculated from MaxEnt output for ten model replicates. Models were built with species-specific background sampling.

|  | ***Variable*** | ***Permutation Importance (mean)*** | ***SD*** |
| --- | --- | --- | --- |
| **Model: American marten**  **All Variables North only** | Summer Heat Moisture Index | 40.6704 | 3.00687 |
|  | Forest Height | 10.8795 | 2.778655 |
|  | Precipitation as Snow | 10.6985 | 4.349391 |
|  | Spruce Fir Forest | 7.1060 | 0.804539 |
|  | Elevation | 6.2817 | 2.34924 |
|  | Dry Forest | 4.5579 | 1.888975 |
|  | Mixed Conifer Forest | 4.1996 | 1.664563 |
|  | Topographic Position Index | 3.9185 | 1.494702 |
|  | Wet Forest | 3.2207 | 1.01723 |
|  | Distance to Forest Edge | 2.2548 | 1.006856 |
|  | Slope | 1.1146 | 0.425452 |
|  | Distance to water | 0.3364 | 0.399793 |
|  | Riparian | 0.2242 | 0.264803 |
|  | Area Burned | 0.2161 | 0.227426 |
|  | Lodgepole Forest | 0.1176 | 0.12732 |
|  |  |  |  |
| **Model: Pacific marten**  **All Variables South only** | Slope | 19.521 | 2.145 |
|  | Precipitation as Snow | 12.463 | 4.330 |
|  | Lodgepole Forest | 11.511 | 1.671 |
|  | Distance to Forest Edge | 10.141 | 3.151 |
|  | Mixed Conifer Forest | 9.227 | 2.663 |
|  | Elevation | 7.286 | 1.989 |
|  | Topographic Position Index | 6.538 | 0.834 |
|  | Summer Heat Moisture Index | 4.449 | 0.854 |
|  | Area Burned | 4.312 | 1.044 |
|  | Spruce Fir Forest | 3.855 | 0.776 |
|  | Forest Height | 3.626 | 1.055 |
|  | Wet Forest | 2.169 | 1.206 |
|  | Dry Forest | 2.01 | 1.090 |
|  | Riparian | 1.795 | 0.659 |
|  | Distance to water | 1.097 | 0.421 |
|  |  |  |  |

**Figure S1:** Results from NewHybrids analysis without priors. Panel is facetted by assignment of parental cluster in STRCUTURE and color coding indicates the probability of assignment to specific hybrid classes in NewHybrids. Samples are ordered latitudinally within facets.


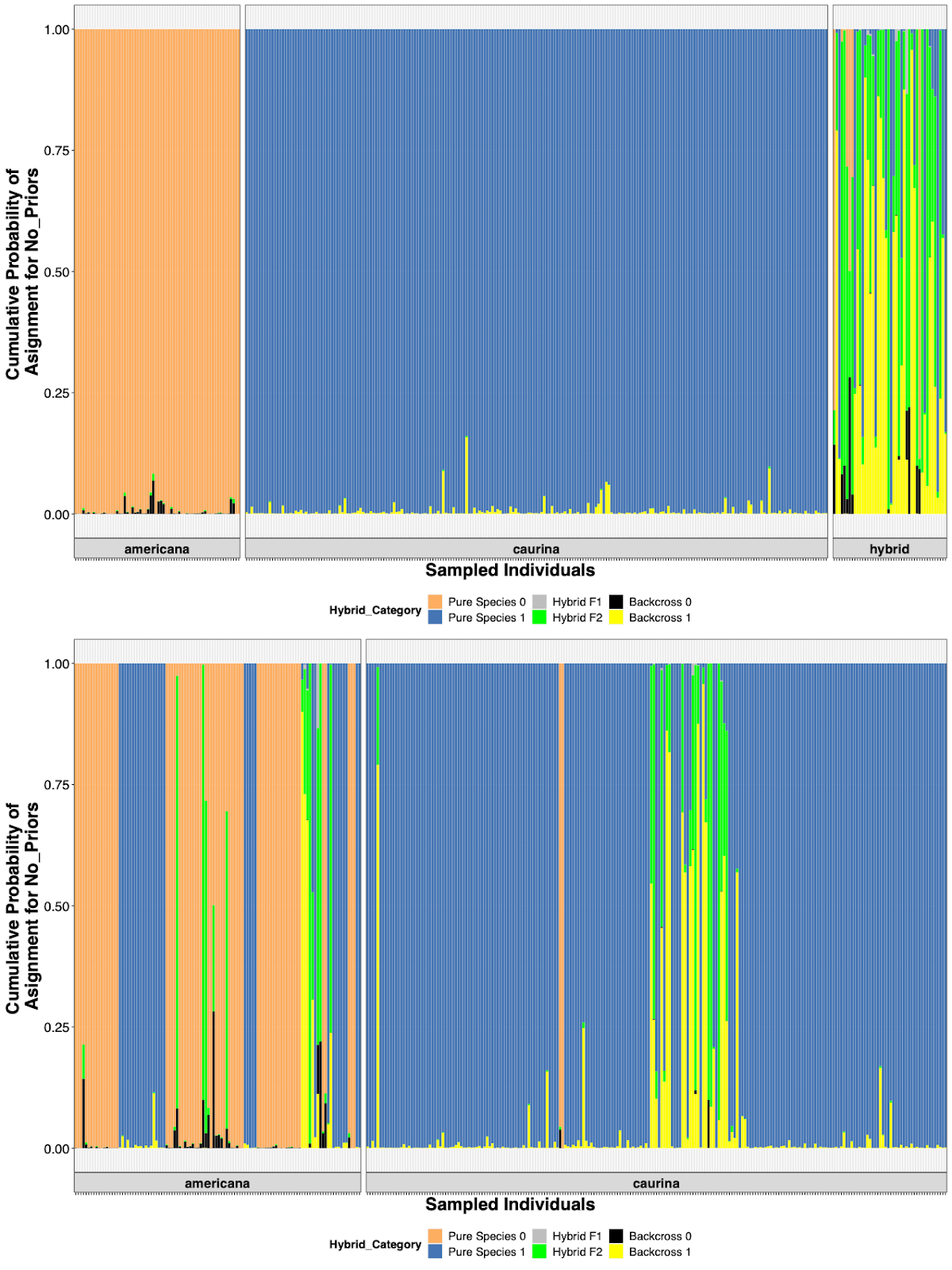


**Figure S2:**  Results from New Hybrids with inclusion of priors. Panel is facetted by assignment of parental cluster in STRCUTURE and color coding indicates the probability of assignment to specific hybrid classes in NewHybrids. Samples are organized latitudinally within facets.


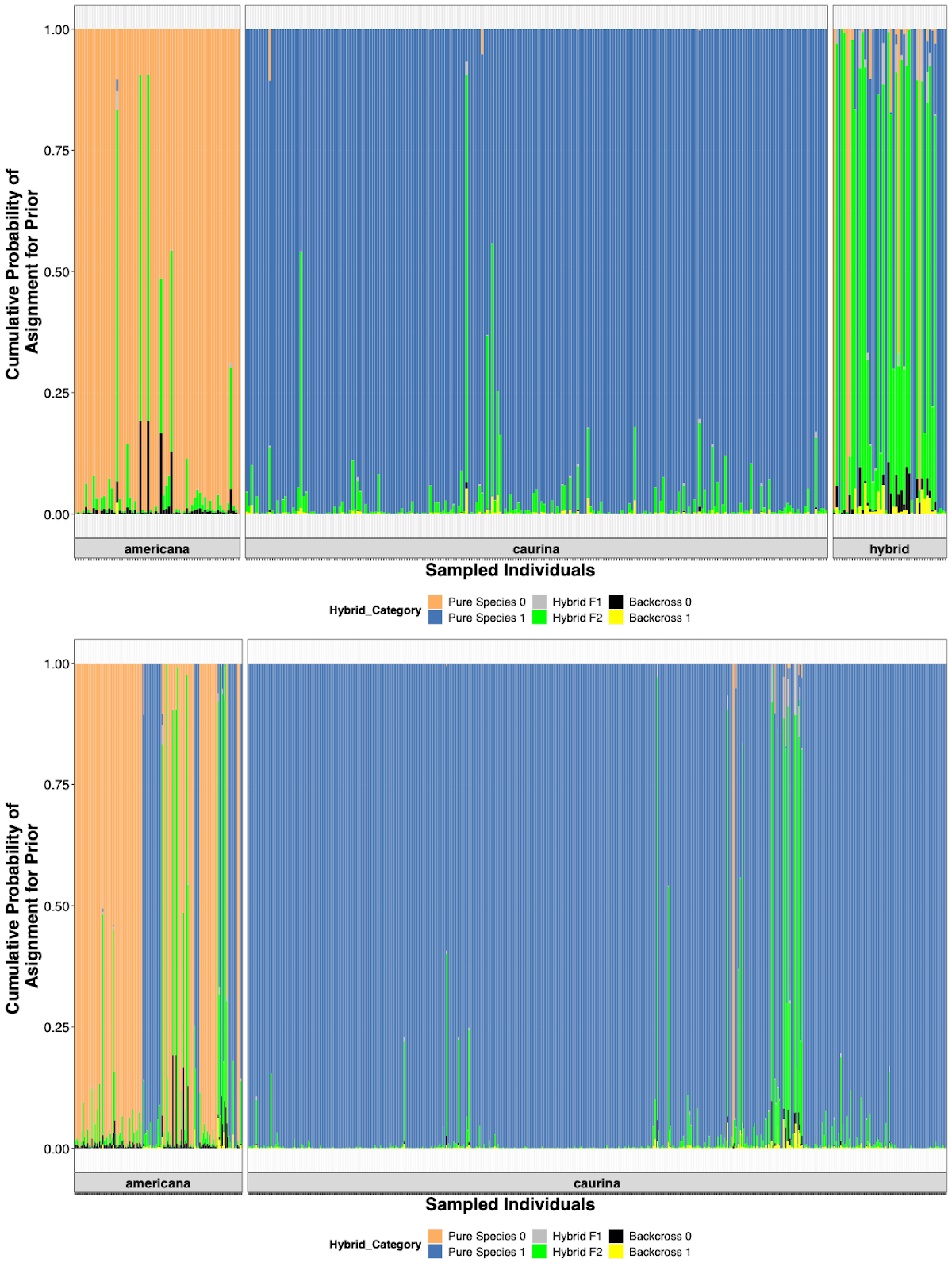


**Figure S3:** American marten (A) and Pacific marten (B) habitat suitability predictions showed some variability depending upon the rules used for generated pseudoabsence background (bg) samples. Color coding in each figure compares habitat predictions across the two background sampling approaches.


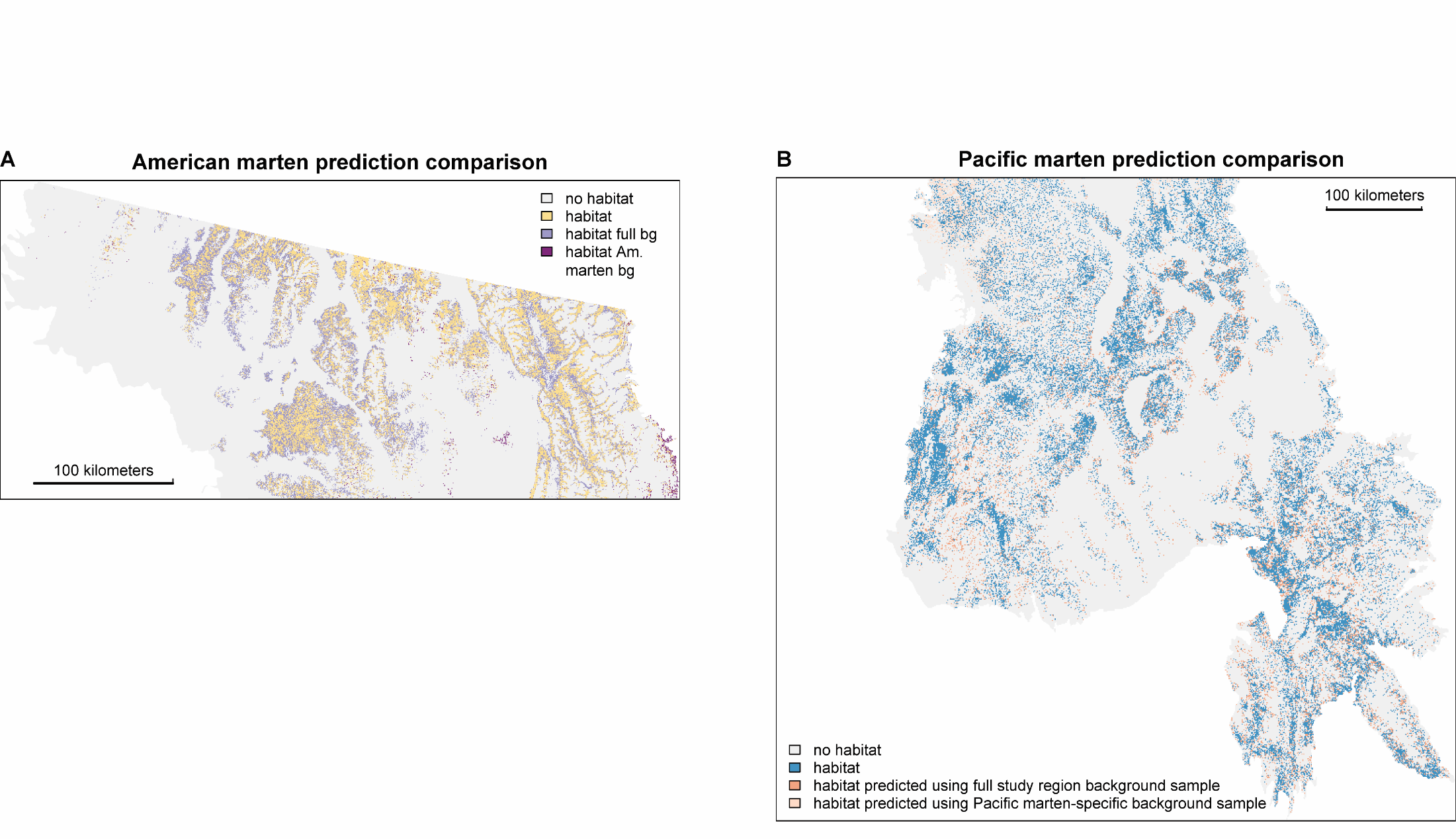


**Figure S4:** Models built using all variables and with climate and topographical variables only (for the purposes of projecting future species distribution) largely agreed on the assignment of suitable vs. unsuitable habitat for the two species. For American marten (A), models agreed on the classification of suitable or unsuitable habitat 96% of the time. Of the total area identified as suitable habitat in at least one of the two models (10% of the study region), 62% was identified as suitable by both models, 15% was identified as suitable in the full model only, and 23% was identified as suitable in the model with only climate and topographical variables. Visual inspection of the results suggests there was more likely to be disagreement between the models in the southern extreme and eastern extreme of the study region. For Pacific marten (B), models agreed in the classification of suitable vs. unsuitable habitat 85% of the time. Of the total area identified as suitable habitat by either model (29% of the study region), 50% was identified as suitable in both models, 15% was identified as suitable in the full model only, and 35% was identified as suitable in the climate and topographic variable model only. Visual inspection of results suggests that the climate and topographical variable model for Pacific marten predicted more habitat in the southeastern portion of the study region than was predicted by the full model.


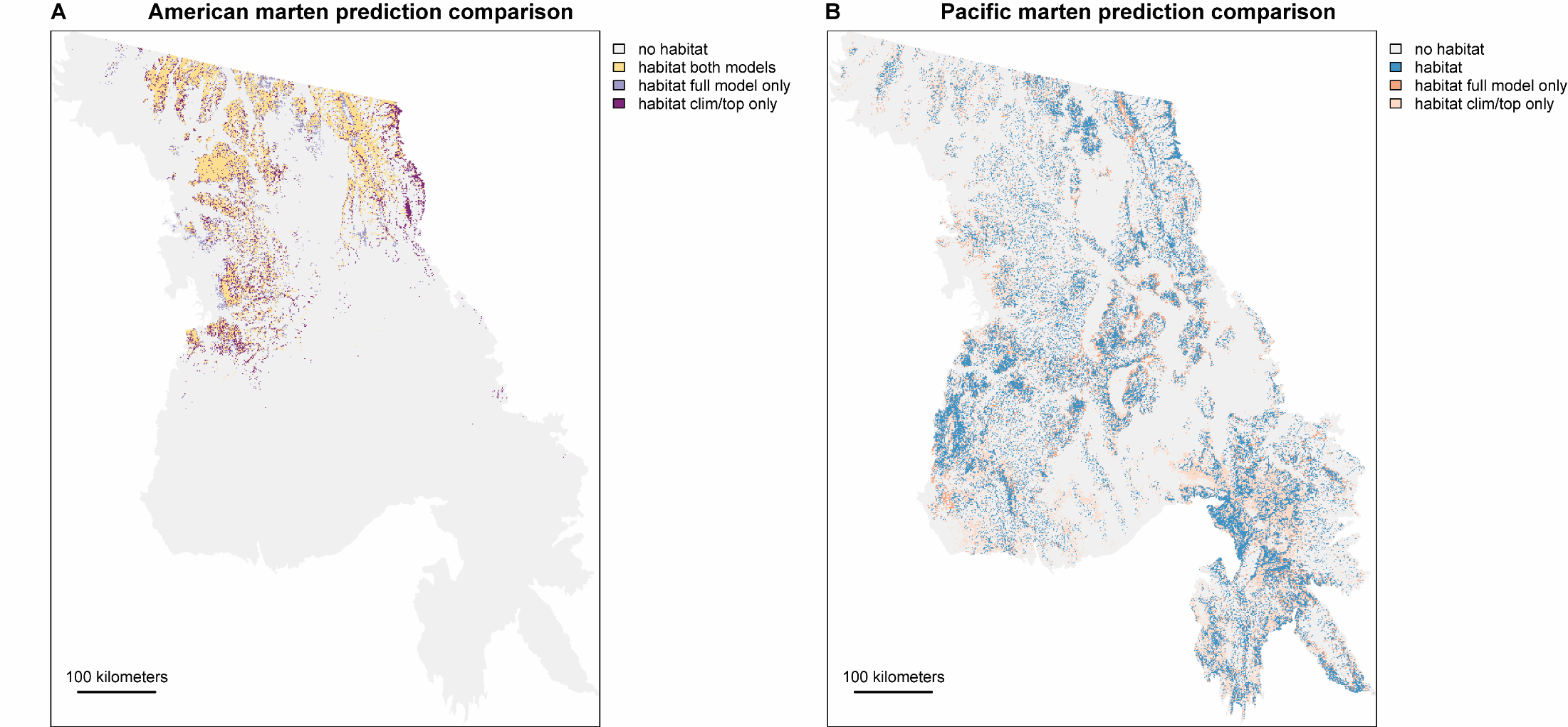


**Figure S5:** Maps depicting predicted marten distribution for the period 2041-2070 by species (A-C: American marten, D-E: Pacific marten) under low, medium, and high emissions scenarios. Color coding represents degree of predicted habitat suitability on a cloglog scale above a minimum threshold maximizing sensitivity and specificity. Maps showing predicted habitat losses and gains are provided in Figure 3.

**
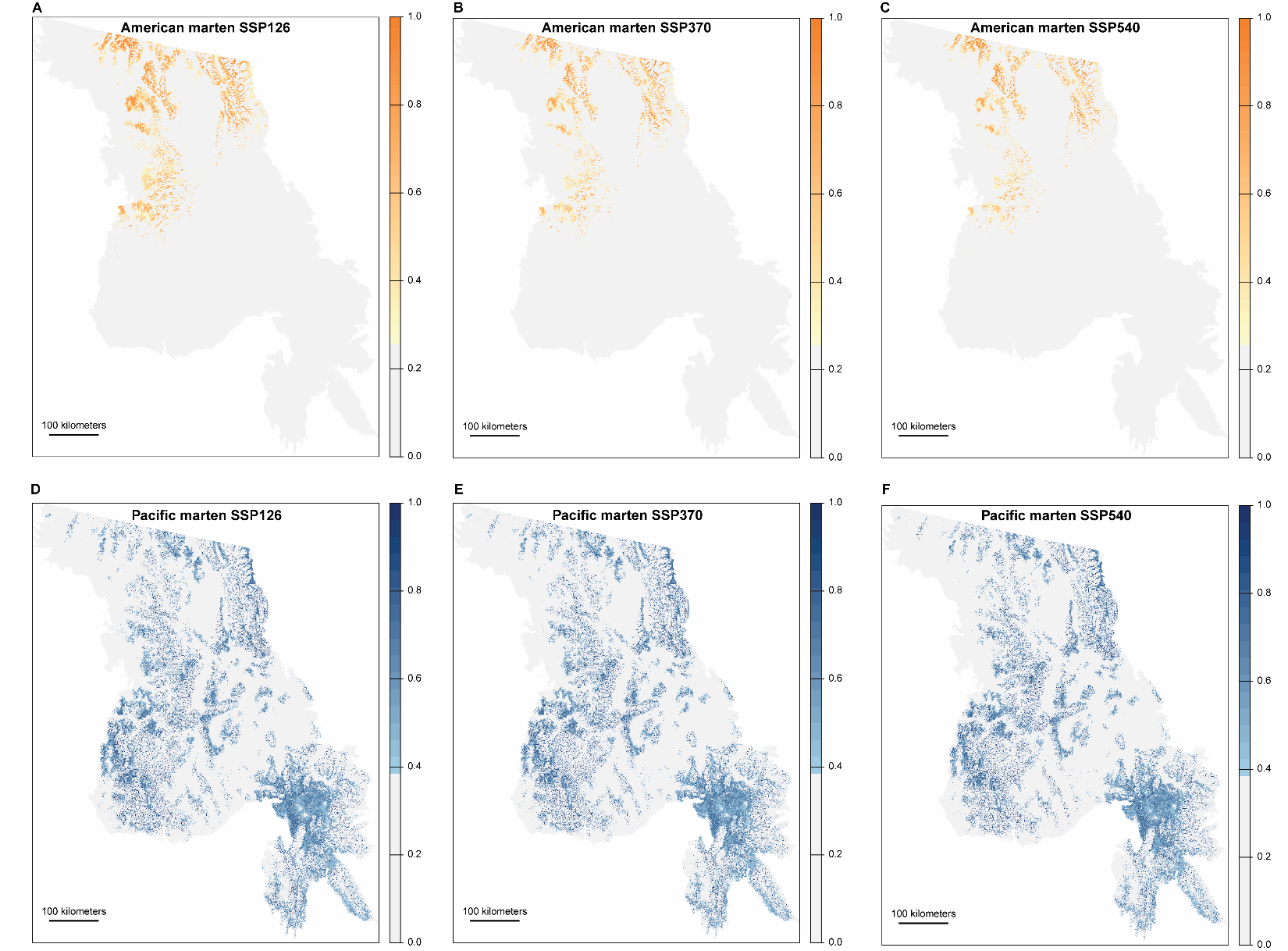
**

**Figure S6:** Marginal response curves for American marten habitat modeled across the full study region with all covariates. Marginal response curves were generated holding the non-focal covariates at a median value.

**
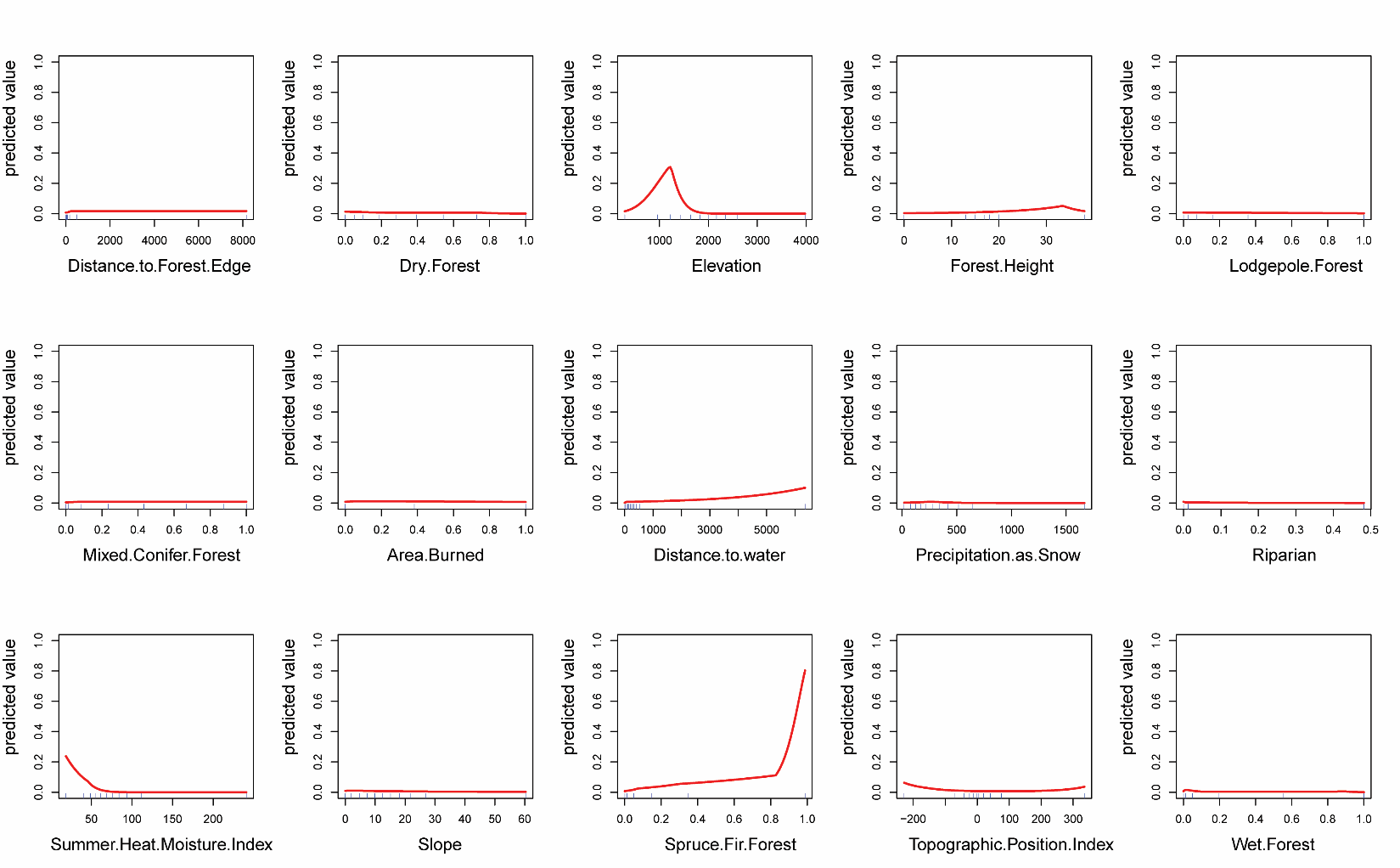
**

**Figure S7:** Marginal response curves for Pacific marten habitat modeled across the study region with all covariates. Marginal response curves were generated holding the non-focal covariates at a median value.


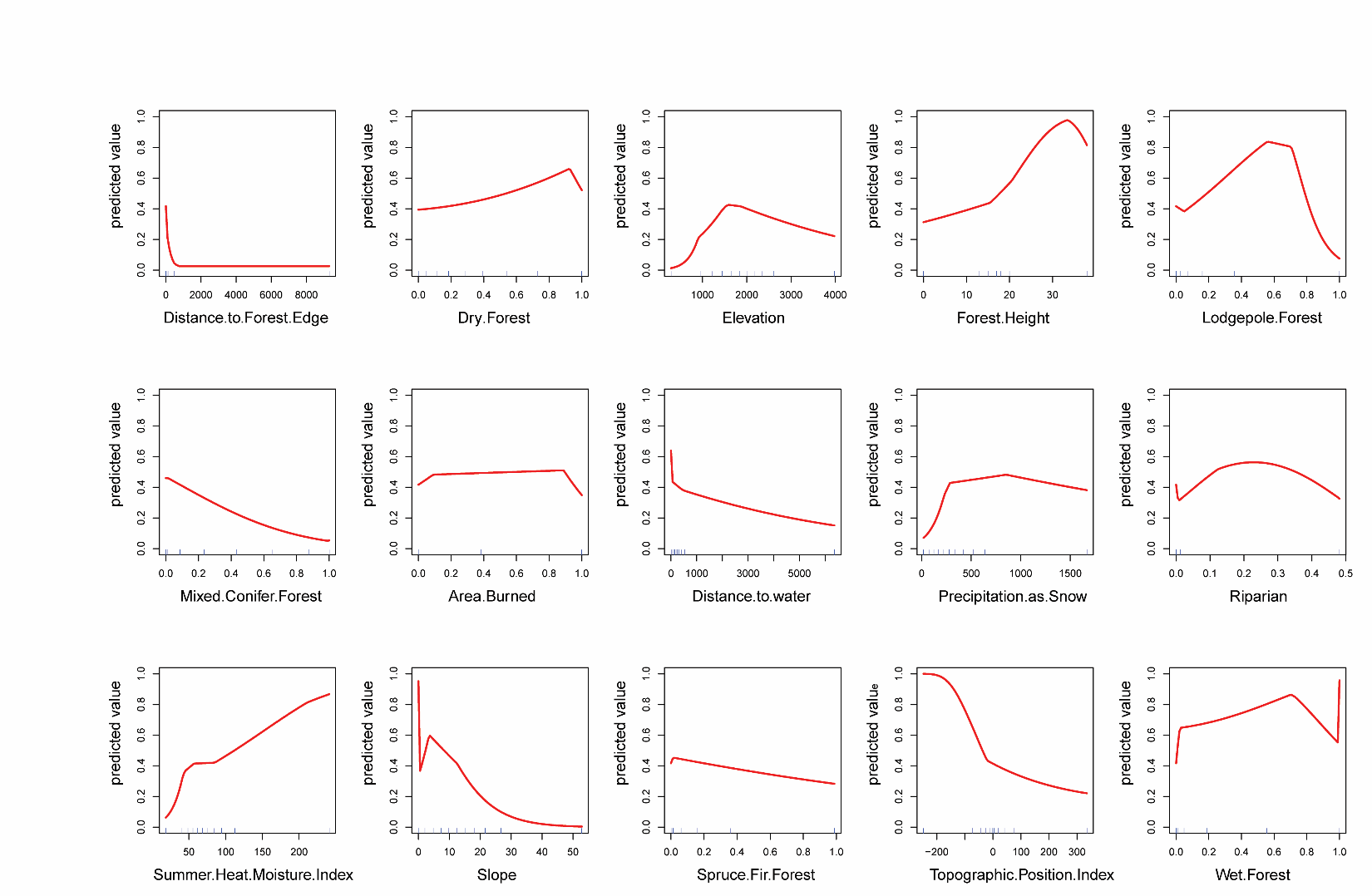

Supplement: Supplementary file 1 — Appendix S1. [file ECE3-14-e70181-s001.docx]
